# Limb Salvage Outcomes Following Endovascular Revascularization for Peripheral Artery Disease in a Southern Indian Population: A Single-Center Retrospective Study

**DOI:** 10.7759/cureus.117715

**Published:** 2026-10-10

**Authors:** Sibasankar Dalai, Aravind Varma Datla, Ananta Rao Baratam, Mohan Rao Ambati, Rajasekhar Donkada, Surendra N Velamala, Chittaranjan N Vangapandu, Rama Sravya Saripalli, Merlin A Abraham

**Affiliations:** 1 Interventional Neuroradiology, Medicover Hospitals, Visakhapatnam, IND; 2 Internal Medicine, Medicover Hospitals, Visakhapatnam, IND; 3 General Surgery, Medicover Hospitals, Visakhapatnam, IND; 4 Critical Care Medicine, Medicover Hospitals, Visakhapatnam, IND; 5 Anaesthesiology, Medicover Hospitals, Visakhapatnam, IND; 6 Emergency Medicine, Medicover Hospitals, Visakhapatnam, IND; 7 Statistics, Medicover Hospitals, Visakhapatnam, IND; 8 Interventional Radiology, Medicover Hospitals, Visakhapatnam, IND

**Keywords:** below-the-knee angioplasty, chronic limb-threatening ischemia (clti), diabetes mellitus, endovascular revascularization, endovascular therapy, india, limb salvage, peripheral artery disease (pad)

## Abstract

Background

Peripheral artery disease (PAD), particularly chronic limb-threatening ischemia (CLTI), is associated with a high risk of limb loss and significant morbidity. Endovascular therapy (ET) has emerged as a minimally invasive alternative to surgical revascularization, especially in high-risk patients and those with comorbidities. This study aims to evaluate the effectiveness of ET in achieving limb salvage in a Southern Indian population.

Materials and methods

This retrospective observational study was conducted at a tertiary vascular care center and included 142 patients with symptomatic PAD who underwent endovascular intervention between July 2023 and June 2025. Clinical severity and lesion complexity were assessed using the Rutherford classification and the TransAtlantic Inter-Society Consensus II (TASC II) classification, respectively. The primary endpoint was limb salvage at one year, defined as freedom from major amputation. Secondary endpoints included technical success, restenosis, and procedure-related complications.

Results

The study population was predominantly male (87.3%) with a mean age of 61.11 ± 12.90 years. Most patients (78.1%) presented with CLTI. Technical success was achieved in 87.3% of procedures. At one-year follow-up, limb salvage was achieved in 95.8% of patients, with major amputation occurring in 4.2% and minor amputation in 9.1%. Restenosis was observed in 14.7% of cases. Procedure-related complications were infrequent and mostly minor, with one all-cause death during follow-up.

Conclusions

ET is a safe and effective modality for limb salvage in PAD patients, particularly in populations with a high prevalence of diabetes and advanced disease at presentation. High limb salvage rates can be achieved even in cohorts predominantly comprising patients with CLTI, supporting an endovascular-first approach in appropriately selected patients.

## Introduction

Peripheral artery disease (PAD) is a chronic, progressive atherosclerotic disorder characterized by partial or complete obstruction of the arterial lumen, most commonly affecting the lower extremities. It is a major global health burden, affecting about 237 million people worldwide and contributing significantly to morbidity, mortality, and the risk of limb loss [1-3]. Despite advances in diagnosis and management, PAD remains under-recognized and undertreated, particularly in low- and middle-income countries.

Atherosclerosis accounts for nearly 95% of PAD cases, leading to progressive luminal narrowing, reduced distal perfusion, and tissue ischemia. Clinically, PAD encompasses a spectrum ranging from intermittent claudication to chronic limb-threatening ischemia (CLTI), the latter being characterized by rest pain, non-healing ulcers, or gangrene and associated with a high risk of amputation if left untreated [3,4]. Furthermore, PAD is a manifestation of systemic atherosclerosis and is associated with an increased risk of major adverse cardiovascular events, including myocardial infarction, stroke, and cardiovascular mortality [4].

Management strategies for PAD focus on symptom relief, limb preservation, and cardiovascular risk reduction, incorporating lifestyle modification, pharmacotherapy, and revascularization. Timely revascularization is particularly critical in CLTI to prevent tissue loss and improve limb salvage outcomes [1,2]. While surgical bypass has traditionally been considered the gold standard for revascularization, it is associated with higher perioperative morbidity and may not be suitable for high-risk or elderly patients [4].

Over the past two decades, endovascular therapy (ET) has emerged as a less invasive alternative, offering lower procedural risk, shorter hospital stays, and faster recovery. Advances in endovascular techniques now allow treatment of complex and distal lesions, including below-the-knee and pedal vessels, with encouraging outcomes in limb salvage and wound healing [5,6]. Recent guidelines from the American College of Cardiology/American Heart Association and the European Society of Cardiology emphasize the role of timely revascularization in CLTI, with increasing acceptance of endovascular-first strategies in appropriately selected patients [1,2,4,7,8].

In the Indian context, PAD often presents at a more advanced stage due to delayed diagnosis, a high prevalence of diabetes, and limited access to specialized vascular care [9]. Patients frequently present with CLTI, extensive distal disease, and increased risk of limb loss, posing unique challenges in management and outcomes. Despite the growing adoption of ET, region-specific data evaluating its effectiveness in Indian populations remain limited.

This study aims to evaluate clinical outcomes, procedural success, and limb salvage rates after ET for PAD in a Southern Indian population at a single tertiary care center, contributing to the limited regional evidence and informing clinical practice in similar settings.

## Materials and methods

This retrospective observational study was conducted at a tertiary vascular care center in a Southern Indian population to evaluate the safety and efficacy of ET for limb salvage in patients with PAD. The study included patients treated between July 2023 and June 2025. A total of 142 patients were identified through a review of the Hospital Information System and Electronic Medical Records.

Adult patients (≥18 years) with symptomatic PAD, ranging from intermittent claudication to CLTI, were included. CLTI was defined as the presence of ischemic rest pain, non-healing ulcers, or gangrene. Patients with acute limb ischemia, non-atherosclerotic arterial disease, or incomplete clinical data were excluded.

Baseline data included demographic characteristics, comorbidities, and clinical severity, stratified according to the Rutherford classification and the TransAtlantic Inter-Society Consensus II (TASC II) lesion classification [10,11]. Pre-procedural imaging included duplex ultrasound and/or angiography extending from the infrarenal aorta to distal runoff vessels.

Endovascular interventions included plain balloon angioplasty, drug-coated balloon angioplasty, bare-metal stent placement, drug-eluting stents, stent grafts, atherectomy, and intravascular lithotripsy for complex lesions. Device selection was based on lesion characteristics, anatomical considerations, and institutional practice protocols. Special emphasis was placed on achieving adequate distal perfusion, with targeted below-the-knee and pedal arch revascularization performed when necessary.

The primary endpoint was limb salvage at one year, defined as freedom from major amputation (above the ankle) in the treated limb. Secondary endpoints included technical success, restenosis, and procedure-related complications. Technical success was defined as restoration of inline flow to the distal vasculature with ≤20% residual stenosis on final angiography.

All procedure-related complications were assessed. Follow-up was conducted at regular intervals (one, three, six, and 12 months) through clinical examination and, where indicated, duplex ultrasound to assess patency and wound healing. Continuous variables were expressed as mean ± standard deviation and categorical variables as percentages. The Institutional Ethics Committee of Medicover Hospitals approved the study protocol (approval number: MH/2026/IEC/June/07), and patient confidentiality was maintained throughout the study.

## Results

During the study period, 142 patients with PAD underwent endovascular intervention, involving the treatment of 317 vascular segments. The study population was predominantly male (87.3%, n = 124), with females comprising 12.7% (n = 18). The mean age was 61.11 ± 12.90 years. Most patients were aged 50-70 years (60.6%, n = 86), followed by those older than 70 years (21.8%, n = 31), while 17.6% (n = 25) were younger than 50 years (Table 1).

**Table 1:** Baseline characteristics of the study population (n = 142)

Variable	Number (%)
Age <50 years	25 (17.6%)
Age 50-70 years	86 (60.6%)
Age >70 years	31 (21.8%)
Male sex	124 (87.3%)
Female sex	18 (12.7%)
Diabetes mellitus	80 (56.3%)
Hypertension	88 (61.9%)
Smoking history	55 (38.7%)
Dyslipidemia	45 (31.6%)
Chronic kidney disease	24 (16.9%)
Coronary artery disease	38 (26.7%)
Cerebrovascular disease	15 (10.5%)

Comorbidities were common, with hypertension present in 61.9% (n = 88) of patients, diabetes mellitus in 56.3% (n = 80), smoking history in 38.7% (n = 55), dyslipidemia in 31.6% (n = 45), chronic kidney disease in 16.9% (n = 24), coronary artery disease in 26.7% (n = 38), and cerebrovascular disease in 10.5% (n = 15).

Most patients presented with advanced disease. Rutherford category 5 was most common (45.0%, n = 64), followed by category 4 (19.8%, n = 28) and category 3 (13.4%, n = 19). Categories 2 and 6 accounted for 8.4% (n = 12) and 13.4% (n = 19), respectively. Overall, 78.1% of patients presented with CLTI (Table 2).

**Table 2:** Disease severity and lesion characteristics (n = 142) CLTI: chronic limb-threatening ischemia, TASC II: TransAtlantic Inter-Society Consensus II

Variable	Number (%)
Rutherford category 2	12 (8.4%)
Rutherford category 3	19 (13.4%)
Rutherford category 4	28 (19.8%)
Rutherford category 5	64 (45.0%)
Rutherford category 6	19 (13.4%)
CLTI (Rutherford 4-6)	111 (78.1%)
TASC II A	30 (21.1%)
TASC II B	49 (34.5%)
TASC II C	41 (28.9%)
TASC II D	22 (15.5%)

The TASC II classification revealed a predominance of intermediate-to-complex lesions (Table 2). TASC II type B lesions were most frequent (34.5%, n = 49), followed by type C (28.9%, n = 41), type A (21.1%, n = 30), and type D (15.5%, n = 22).

Among the treated vascular segments (n = 317), the superficial femoral artery was the most commonly treated vessel (40.4%, n = 128), followed by below-the-knee vessels (23%, n = 73) and the popliteal artery (21.4%, n = 68). Iliac artery interventions accounted for 9.8% (n = 31), while common femoral and aortic interventions were infrequent (Table 3).

**Table 3:** Distribution of treated vascular segments (n = 317)

Vascular segment	Number (%)
Superficial femoral artery	128 (40.4%)
Popliteal artery	68 (21.4%)
Below-the-knee vessels	73 (23%)
Iliac artery	31 (9.8%)
Common femoral artery	11 (3.5%)
Aorta	6 (1.9%)

Technical success was achieved in 124 of 142 patients (87.3%). At one-year follow-up, limb salvage was achieved in 95.8% of patients despite a predominantly CLTI population. Major amputation occurred in 4.2% (n = 6), while minor amputations were required in 9.1% (n = 13). Mortality during follow-up was low, with one all-cause death occurring during the one-year follow-up period (0.7%); the cause of death could not be ascertained from the available records. Restenosis within one year was observed in 14.7% (n = 21) of cases (Table 4).

**Table 4:** Procedural and clinical outcomes

Outcome	Number (%)
Technical success	124 (87.3%)
Limb salvage at one year	136 (95.8%)
Major amputation	6 (4.2%)
Minor amputation	13 (9.1%)
All-cause mortality at one year	1 (0.7%)
Restenosis at one year	21 (14.7%)

Procedure-related complications were observed in a minority of patients. Access-site complications included hematoma in 6.3% (n = 9) and pseudoaneurysm in 1.4% (n = 2). Vascular complications included vessel dissection (3.5%, n = 5), perforation (8.4%, n = 12), rupture (1.4%, n = 2), acute thrombosis (3.5%, n = 5), and distal embolization (2.1%, n = 3). Device-related complications were infrequent, with stent thrombosis in 2.1% (n = 3) and stent fracture in 0.7% (n = 1). Contrast-induced nephropathy occurred in 3.5% (n = 5). Most complications were minor and managed conservatively (Table 5).

**Table 5:** Complications

Complications	Number (%)
Access site hematoma	9 (6.3%)
Pseudoaneurysm	2 (1.4%)
Vessel dissection	5 (3.5%)
Vessel perforation	12 (8.4%)
Vessel rupture	2 (1.4%)
Acute thrombosis	5 (3.5%)
Distal embolization	3 (2.1%)
Stent thrombosis	3 (2.1%)

## Discussion

PAD, particularly CLTI, is associated with a high risk of limb loss and poor quality of life [1-9]. In our study, we evaluated the role of ET in achieving limb salvage in a Southern Indian population. We observed favorable outcomes, with high rates of technical success and limb preservation. A key finding is a 95.8% limb salvage at one year rate in a predominantly high-risk cohort, with 78.1% of patients presenting with CLTI. CLTI represents the most severe form of PAD and requires timely revascularization to restore perfusion, prevent tissue loss, and reduce mortality [1,3,7,12]. These findings highlight the effectiveness of ET even in advanced disease, particularly in the Indian context, where delayed presentation and a high prevalence of diabetes contribute to more severe disease at diagnosis [9,13,14]. ET has emerged as an important revascularization strategy because it is minimally invasive, has lower peri-procedural morbidity, and can treat complex and distal lesions [3,4,6,12,15]. Contemporary guidelines, including those from the American College of Cardiology/American Heart Association and the Global Vascular Guidelines, support timely revascularization and increasingly favor an endovascular-first approach in appropriately selected patients [1,2,4-8,16].

In our cohort, a substantial proportion of interventions involved below-the-knee and distal vessels, emphasizing the importance of achieving adequate distal perfusion. Restoration of inline flow to the foot is a critical determinant of wound healing and limb salvage [3,4,6].

The epidemiology and clinical presentation of PAD in the Indian population differ from those in Western cohorts. Although the overall prevalence of PAD may be lower, patients often present at a younger age and with more advanced disease, particularly involving distal infrapopliteal vessels [9,13,14,17,18]. This pattern is largely driven by the high prevalence of diabetes mellitus, which was present in over half of our study population. Diabetic PAD is characterized by multilevel and distal disease, microvascular dysfunction, and impaired wound healing, making endovascular approaches particularly suitable for revascularization in this subset [15,19].

Our findings are consistent with contemporary evidence supporting the effectiveness of ET in limb salvage. Large observational studies and registries have demonstrated high limb salvage rates with acceptable safety profiles [8,12]. The BEST-CLI trial showed that surgical bypass may be superior in patients with an adequate single-segment great saphenous vein conduit; however, in patients without suitable conduits, ET outcomes were comparable [20]. In contrast, the BASIL-2 trial, which focused on infrapopliteal disease, showed improved amputation-free survival with ET compared to bypass surgery [21]. These findings are particularly relevant to our cohort, which demonstrated a high burden of distal disease, supporting the applicability of an endovascular-first strategy in similar populations.

Meta-analyses and registry-based data further support the favorable peri-procedural safety profile of ET, with lower rates of major adverse cardiac events, bleeding, and wound complications, as well as shorter hospital stays compared to surgical revascularization [15,22]. The LIBERTY study and Vascular Quality Initiative registry have similarly shown lower morbidity and acceptable limb-salvage outcomes with endovascular interventions in real-world settings [23,24]. Additionally, the ability to perform repeat or staged interventions offers a practical advantage, particularly for patients with prolonged wound healing [18].

In our study, complication rates were low and predominantly minor. One all-cause death occurred during follow-up (0.7%); no procedure-related mortality was observed. These findings align with the literature demonstrating the safety of endovascular approaches [15,16,18]. However, restenosis remains an important limitation, with a 14.7% restenosis rate at one year. This highlights the need for ongoing surveillance and, where necessary, repeat interventions to maintain vessel patency.

This study's findings support an individualized, patient-centered approach to revascularization that accounts for anatomical complexity, comorbidities, and conduit availability. In resource-constrained settings and high-risk populations, ET offers a practical and effective strategy for limb salvage.

The retrospective, single-center design of this study may limit the generalizability of the findings. The relatively short follow-up period restricts assessment of long-term patency and durability. Additionally, the absence of a comparative surgical cohort limits direct comparison between treatment modalities. Future prospective, multicenter studies with longer follow-up are needed to validate these findings.

## Conclusions

ET is a safe and effective modality for limb salvage in patients with PAD, particularly in populations with a high prevalence of diabetes and advanced disease at presentation. High limb salvage rates can be achieved even in predominantly CLTI cohorts, supporting an endovascular-first approach in appropriately selected patients.
